# Discovery of small molecules with anthelmintic potential in the Medicines for Malaria Venture’s COVID and Global Health Priority Boxes using an infrared-based assay for *Caenorhabditis elegans* motility

**DOI:** 10.1186/s13104-025-07485-9

**Published:** 2025-10-06

**Authors:** Yujie Uli Sun, Lawrence J. Liu, Conor R. Caffrey

**Affiliations:** Center for Discovery and Innovation in Parasitic Diseases, Skaggs School of Pharmacy and Pharmaceutical Sciences, University of California San Diego, 9255 Pharmacy Lane, MC0657, La Jolla, CA 92093 USA

**Keywords:** Whole-organism screen, *Caenorhabditis elegans*, Anthelmintic, Drug discovery, Parasite, Medicines for Malaria Venture

## Abstract

**Background:**

Parasitic nematodes are a public health problem globally, and an economic burden on animal and plant agricultural industries. With their ability to generate drug resistance, new anthelmintic compounds must be constantly sourced.

**Methods:**

Using the free-living nematode, *Caenorhabditis elegans*, in an infrared-based motility assay, we screened 400 compounds from two open-source, small-molecule collections distributed by the Medicines for Malaria Venture, namely, the COVID Box and Global Health Priority Box. The screening assay was first validated for worm number, DMSO concentration and final volume.

**Results:**

Primary and secondary (time- and concentration-dependent) screens of both boxes, identified twelve compounds as hits; nine of which are known anthelmintics. Three additional bioactives, flufenerim, flucofuron and indomethacin were identified with EC_50_ values ranging from 0.211 to 23.174 µM. Counter toxicity screens with HEK293 cells indicated varying degrees of toxicity with EC_50_ values ranging from 0.453 to > 100 µM.

**Conclusions:**

A *C. elegans* motility assay was optimized and used to screen two recently-released, small molecule libraries. Flufenerim, flucofuron and/or indomethacin might serve as starting points for the development of new anthelmintics.

**Supplementary Information:**

The online version contains supplementary material available at 10.1186/s13104-025-07485-9.

## Introduction

Parasitic nematodes infect more than one quarter of the global population [1, 2] and promote poverty by constraining personal and societal productivity [2, 3]. Just a handful of drug classes are available [4], and failed treatments are known [5–7]. Nematode parasites are also an economic burden to agriculture [8–10] with drug resistance being quick to emerge [11]. Accordingly, new drugs are urgently needed.

The free-living nematode, *Caenorhabditis elegans*, is useful in anthelmintic drug discovery [12–15]. We developed and deployed a *C. elegans* screen to evaluate two small molecule ‘box’ collections released by the drug development consortium, the Medicines for Malaria Venture (MMV). These COVID and Global Health Priority (GHP) Boxes, comprise 160 and 240 compounds, respectively, and contain bioactives for the SARS-CoV-2 virus, and various pathogens and vectors, respectively. Both boxes are the latest in a series of boxes distributed *gratis* by the MMV to spur drug discovery for infectious diseases [16–24].

## Methods

### Chemicals

The COVID and GHP Boxes were provided by the MMV, Geneva, Switzerland as 10 µL solutions in DMSO (10 or 2 mM) in 96-well polypropylene plates. Plates were stored at – 80 °C.

Ivermectin (Cat#: I8898), doramectin (Cat#: 33993), selamectin (Cat#: SML2663) and tolfenpyrad (Cat#: 37043) were from Sigma Aldrich. Milbemectin (Cat#: 22003) and indomethacin (Cat#: 70270) were from Cayman Chemical, and moxidectin (Cat#: J92012), abamectin (Cat#: I019), chlorfenapyr (Cat#: J96076) and eprinomectin (Cat#: 7712CY) were from AK Scientific. Flucofuron (Cat#: CS-0009766) was from ChemScene.

### C. elegans maintenance and motility assay

Methods to cultivate *C. elegans* (Bristol N2) and synchronize growth to the L4 stage were as described [25]. WMicroTracker ONE (Phylumtech, Argentina) detects movement by measuring the scattering of infrared light beams that are projected into each well of a microtiter plate (two 880 nm beams/well in a 96w plate) [26].

Synchronized *C. elegans* L4 were detached from agar and collected in M9 buffer. Worms were centrifuged for 1 min at 1,900 *g* and washed in S medium to decrease the concentration of *E. coli* OP50 that might interfere with infrared detection. Control and assay compounds (1 µL) in DMSO (Fisher Scientific, Cat# BP231-100) were spotted into each well of a clear, flat-bottomed 96-well polystyrene plate (Fisherbrand, Cat# FB012931). DMSO (1%) was used as the negative control. Approximately 70 L4 in 100 µL S medium were added per well. First pass screens employed 40 µM compound, and motility was measured every 20 min for 24 h in the WMicroTracker ONE reader in which the temperature was 25 ± 1 °C. Motility was normalized relative to the DMSO controls. Hits were defined as compounds that decreased motility to ≤ 25% of that in DMSO control worms. Data for all of the compounds screened are presented in Additional Table 1.

For potent compounds, concentration response assays (nine concentrations: 0.005 µM to 40 or 100 µM) were conducted to measure the half-maximal effective concentration (EC_50_). Compounds were serially diluted in DMSO using 96-well, v-bottomed polypropylene dilution plates (ThermoFisher Scientific, Cat#: 249944) and 1 µL aliquots spotted into each well of the assay plates. Motility was measured as described above. Prism GraphPad, Version 8.0 (GraphPad Software, San Diego, CA) was used to calculate EC_50_ values using a non-linear sigmoidal four parameter logistic curve.

### Human embryonic kidney (HEK) 293 cytotoxicity assay

Potent compounds were also evaluated for cytotoxicity against HEK293 cells. Cells were cultured at 37 °C and 5% in Dulbecco’s Modified Eagle Medium (DMEM; Gibco, Cat#: 11965-092) containing 10% heat-inactivated fetal bovine serum (FBS; Omega Scientific Inc., Cat#: FB02) and 1% penicillin-streptomycin (ThermoFisher Scientific, Cat#: 15140122), and then sub-cultured when 60–80% confluent. Cells were detached with 0.05% trypsin/EDTA (ThermoFisher Scientific, Cat#: 25300054) and centrifuged for 5 min at 1,900 *g*.

Screens were performed over 11 compound concentrations (0.00007–40 µM) that had been prepared in DMSO. Aliquots (1 µL) were spotted into each well of a clear-bottomed, 96-well, polystyrene assay plate (Fisher Scientific, Cat#: FB012931). Approximately 20,000 HEK293 cells in 99 µL of the above supplemented DMEM were then added to each well. After 46 h at 37 °C and 5% CO_2_, 20 µL 0.5 mM resazurin (ThermoFisher Scientific, Cat#: B21187.03) were added and the incubation continued for 2 h at 37 °C [27]. Fluorescence was measured in a 2104 EnVision Multilabel Plate Reader (PerkinElmer) at 560 and 590 nm excitation and emission wavelengths, respectively. Raw data were exported from the EnVision Workstation software (version 1.13.3009.1401; PerkinElmer) into Prism GraphPad, and the half-maximal cytotoxic concentration (CC_50_) values were calculated using a non-linear regression curve.

## Results

### WMicroTracker assay optimization

To optimize the WMicroTracker assay, these variables were considered: the number of L4, the presence and concentration of DMSO, and the final assay volume per well.

**Fig. 1:**
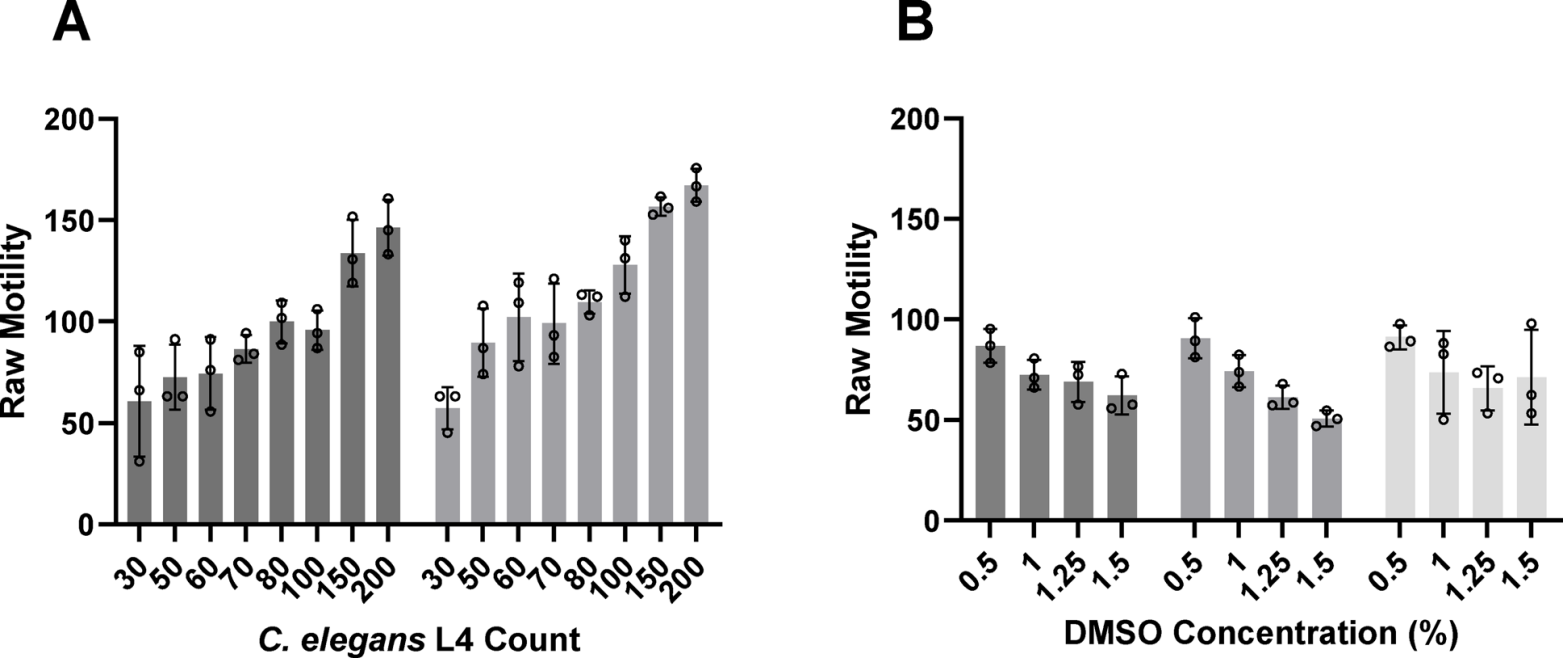
WMicroTracker assay optimization. (**A**) *C. elegans* motility as a function of worm number and the presence (dark grey) or absence (light grey) of 1% DMSO in a final volume of 100 µL. The columns and error bars display the mean ± SD values from one experiment performed in triplicate. A one-sample *t*-test found no difference between 0.5% and 1% DMSO. (**B**) *C. elegans* motility as a function of DMSO concentration and final assay volume. Final volumes from left to right (dark to light grey): 100, 150 and 200 µL. The columns and error bars display the mean ± SD values from one experiment performed in triplicate. One-sample *t*-tests found no difference between 0.5% and 1% DMSO for each of the three volumes tested. For each of the four DMSO concentrations tested, a one-way ANOVA found no difference between the three volumes tested. For both **A** and **B**, motility in raw units was recorded at the 24 h time point

Regarding the number of L4 per well, 30, 50, 60, 70, 80, 100, 150 and 200 L4 were tested in 100 µL S medium in the presence or absence of 1% DMSO. No significant effect was measured with or without DMSO, and for both conditions, motility trended upwards with increasing worm numbers (Fig. 1A). The greatest number of worms (150 or 200) resulted in the highest raw motility units, which would potentially improve the dynamic range of the assay; however, using so many worms would constrain assay throughput. By contrast, there was no statistically significant difference in the motility measured between 70 and 100 worms, and, in the interests of economy, we selected 70 L4 per well.

Due to its thick collagen-enriched cuticle and extensive xenobiotic metabolism [28, 29], *C. elegans* is often exposed to relatively high concentrations of screen compounds. This requires consideration of the final concentration of DMSO to maintain solubility. Therefore, the effect of DMSO concentrations between 0.5 and 1.5% on motility was measured. In parallel, we also considered the final assay volume (100, 150 and 200 µL).

Increasing the DMSO concentration decreased L4 motility in the final volumes of 100 and 150 µL, but less so in 200 µL (Fig. 1B). In all three volumes, motility at 0.5% and 1% DMSO showed no significant difference. Thus, a final concentration of 1% DMSO in 100 µL was chosen to maximize compound solubility in the least assay volume.

### Primary screening of the COVID and GHP Boxes

**Fig. 2:**
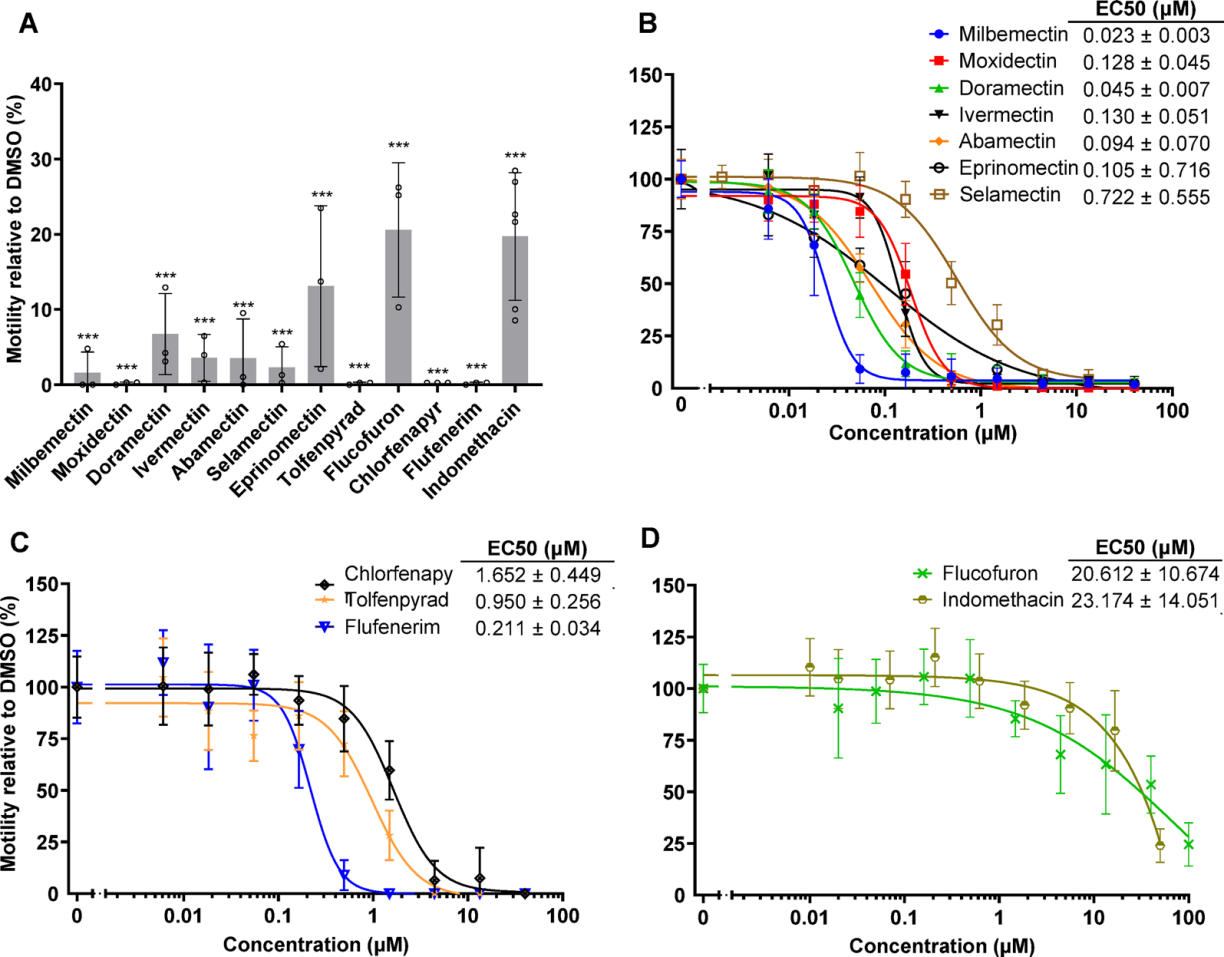
Primary and concentration-response screen data for the 12 primary hits. (**A**) Primary screening identified twelve hits in the MMV COVID and GHP Boxes. *C. elegans* L4 were exposed to 40 µM compound and incubated for 24 h. Motility was normalized to that of the DMSO control. Data displayed represent the means ± SD values from one or two independent experiments, each performed in triplicate. Statistical analysis, comparing to the DMSO control, was conducted using one-way ANOVA with Dunnett’s test: *** = *p* < 0.0001. (**B** – **D**) Concentration response data for the 12 primary hits. L4 were incubated with nine concentrations of compound, and the data evaluated after 1 h (**B**), 12 h (**C**) or 24 h (**D**) to calculate EC_50_ values. NOTE: data for selamectin (**A**) were taken after 3 h. Shown are the means ± SD values from two or three independent assays, each performed in duplicate

The COVID and GHP Box compounds were screened at 40 µM over 24 h (Additional Table 1). Twelve compounds were potent (motility < 25% of DMSO; Fig. 2A). Seven compounds are established macrocyclic lactone anthelmintics [30]: milbemectin (MMV1578924; 1.69% motility relative to control), moxidectin (MMV1633828; 0.28%), doramectin (MMV1633823; 6.74%), ivermectin (MMV672931; 3.37%), abamectin (MMV1577454; 3.60%), selamectin (MMV002231; 2.31%) and eprinomectin (MMV1633829; 13.19%). Another hit, the insecticide, tolfenpyrad (MMV688934; 0.26%), is a known active against the parasitic nematode, *Haemonchus contortus* [24] and *C. elegans* in screens of the MMV’s Pathogen Box [15]. Tolfenpyrad inhibits the electron transport chain complex I and impairs ATP-production [31]. The identification of the macrocyclic lactones and tolfenpyrad as potent actives in our assay validates its utility to identify anthelmintics.

Of the four remaining hits, chlorfenapyr (MMV1577458; 0.26% motility relative to control), flucofuron (MMV027339; 20.31%), flufenerim (MMV1794206; 0.26%) are pesticides, whereas indomethacin (MMV002813; 18.64%) is a non-steroidal anti-inflammatory drug (NSAID).

### Concentration response screens

To determine the relative potency of the 12 primary hits, concentration response curves were generated. Also evaluated was the time-to-effect, whereby compounds were classified by the time point at which an EC_50_ value was first calculable, namely, after 1 h (Fig. 2B), 12 h (Fig. 2C) or 24 h (Fig. 2D). All macrocyclic lactones, except selamectin, demonstrated EC_50_ values < 1 µM after 1 h. The current EC_50_ value of 0.130 ± 0.051 µM for ivermectin is similar to the value of 0.19 ± 0.01 µM previously recorded after 90 min [13]. Selamectin was somewhat slower acting with an EC_50_ value after 3 h of 0.722 ± 0.555 µM. After 12 h, the EC_50_ values for chlorfenapyr, tolfenpyrad and flufenerim were 1.652 ± 0.449 µM, 0.950 ± 0.256 µM and 0.211 ± 0.034 µM, respectively (Fig. 2C). The value measured here for tolfenpyrad after 12 h was higher than that previously recorded after 24 h (0.20 ± 0.04 µM) [15], but still within one order of magnitude. After 24 h, the EC_50_ values for the remaining two hits, flucofuron and indomethacin, were 20.612 ± 10.674 µM and 23.174 ± 14.051 µM, respectively (Fig. 2D).

### HEK293 cell cytotoxicity assay

**Fig. 3:**
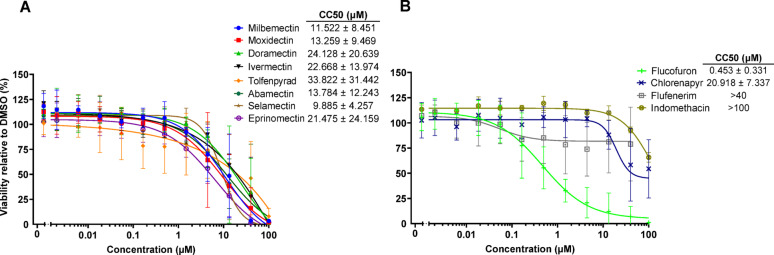
Cytotoxicity of the 12 hit compounds. HEK293 cell viability was measured after 48 h for the seven macrocyclic lactones and tolfenpyrad (**A**), and for flucofuron, chlorfenapyr, flufenerim and indomethacin (**B**). Data represent the means ± SD from two experiments each performed in duplicate

The concentration-dependent cytotoxicity of the 12 hit compounds was measured using HEK293 cells after 48 h (Fig. 3). The macrocyclic lactones and tolfenpyrad were relatively non-toxic with CC_50_ values between 10 and 34 µM (Fig. 3A). Likewise, chlorfenapyr, flufenerim and indomethacin were essentially non-toxic, with CC_50_ values between 21 and > 100 µM. More potent cytotoxicity was measured for flucofuron (0.453 ± 0.331 µM; Fig. 3B).

## Discussion

Parasitic nematode infections are a global health problem and a continual challenge to agricultural productivity [1, 2] due to the emergence and establishment of drug resistance. Thus, new drugs are needed. In this context, we screened the MMV’s recently-released COVID and GHP Boxes against the free-living nematode, *C. elegans*, which has a recognized utility in the identification of anthelmintics [12–15].

We employed the WMicroTracker ONE infrared-based motility device which we preliminarily reported as a useful screening tool [32]. The device has also been employed with various nematode species for drug repurposing and to identify active natural products [33, 34]. It is economical, simple to use and its associated software is easy to learn. Also, the quantitative outputs allow for comparisons of compound efficacy, a notable alternative to manually estimating mortality (e.g [12, 35]), which can be time-consuming and require an understanding of nematode morphology.

After optimizing the assay for worm numbers, DMSO concentration and assay volume, we performed single concentration screens. Among the potent hit compounds were seven established macrocyclic lactone anthelmintics, which were, in the main, fast-acting with sub-micromolar EC_50_ values after 1 h.

Two other hits, tolfenpyrad and chlorfenapyr, were identified with EC_50_ values measurable after 12 h. Of these, chlorfenapyr, a pyrrole class pro-insecticide, was recently employed as a test compound in a screen to measure the response variations among eight *C. elegans* strains to environmental toxicants [36]. In animals, dealkylation of an ethoxymethyl group on chlorfenapyr, reveals the active metabolite, tralopyril, which uncouples mitochondrial oxidative phosphorylation to disrupt ATP-production and cause death [37]. Although relatively non-toxic to HEK293 cells (EC_50_ = 20.918 ± 7.337 µM), there have been poisoning cases with chlorfenapyr [38–40], likely undermining its direct repurposing potential as an anthelmintic.

Our screen also identified three additional bioactives, flufenerim, flucofuron and indomethacin, which were first reported by us in December 2023 in a preprint [41]. During peer-review, another report appeared that employed WMicroTracker ONE to screen *C. elegans* L4 and exsheathed L3 of the nematode parasite, *Haemonchus contortus*, with the GHPB [42]. That report identified flufenerim as a hit against both species, but flucofuron as a hit only against the parasite. The report also confirmed the bioactivity against *C. elegans* of the anthelmintics shown in Fig. 2A and B, with the exception of tolfenpyrad for reasons that are not yet clear.

An EC_50_ value was calculable for flufenerim after 12 h, whereas the values for flucofuron and indomethacin were only calculable after 24 h. The pyrimidinamine, flufenerim, is a potent plant insecticide [43]. Although its mode of action is not confirmed, it inhibits acetylcholinesterase in vitro and in vivo [43]. Acetylcholinesterase is an established anthelmintic/insecticide drug target [44–46] and, if the primary target here, then it may be possible to modify the compound’s structure to enhance specificity and selectivity.

Flucofuron is a bisarylurea pesticide that has been used to mothproof wool [47]. It is a relatively modest inhibitor of worm motility (EC_50_ = 20.612 ± 10.674 µM) with some HEK293 cell toxicity (CC_50_ = 0.453 ± 0.331 µM). In cancer cells, flucofuron and its lipophilic, electron-withdrawing analogues, uncouple mitochondria via a fatty acid-activated mechanism, thereby disrupting ATP-production to cause apoptosis [48]. Disrupting oxidative phosphorylation is a key anti-parasitic strategy [12, 49, 50], and like flufenerim, flucofuron may be chemically modifiable to generate improved specificity for the nematode target, although given the toxicity data recorded here, this may be challenging.

The third hit, the indole-based NSAID, indomethacin, was not cytotoxic to HEK293 cells. Like other NSAIDs, indomethacin inhibits cyclooxygenase (COX) enzymes and their production of prostaglandins, which mediate inflammation [51]. In this context, indomethacin was shown to decrease prostaglandin production and increase parasite burden in mice infected with *Strongyloides venezuelensis* infection [52]. Interestingly, no COX-like enzyme is present in *C. elegans* [53–55], suggesting that indomethacin may act on a novel target(s). COX-2 selective indomethacin analogs with excellent safety profiles have been synthesized [56]. Thus, the opportunity exists to possibly improve on the modest potency demonstrated here for indomethacin *vs. C. elegans* by further screening these or other analogs, combined with genetic approaches to identify the target(s).

## Limitation

*C. elegans* is a proven model for anthelmintic drug discovery [12–15], and two of the three bioactives discovered here have been independently confirmed for a parasitic nematode in vitro [42]. Nonetheless, this in vitro bioactivity still needs to be translated to efficacy in animal models of nematode infection if their anthelmintic potential is to advance.

## Supplementary Information

Below is the link to the electronic supplementary material.

Additional file 1: Additional Table 1. Primary screen activities of the 400 compounds in the MMV COVID and Global Health Priority Boxes. Compounds were tested at 40 µM for modulation of *C. elegans* motility after 24 h. Relative to DMSO control, the degree to which worm motility was decreased was characterized as a hit if < 25%. One (Global Health Priority Box (GHPB)) or two assays (COVID Box) in triplicate was performed. P-values were calculated using a one-sample *t*-test for the hit compounds only.

## Data Availability

No datasets were generated or analysed during the current study.

## Abbreviations

DMEM: Dulbecco’s Modified Eagle Medium
DMSO: Dimethyl sulfoxide
EDTA: Ethylene diamine tetra acetic acid
FBS: Fetal bovine serum
GHP: Global Health Priority
HEK: Human embryonic kidney
MMV: Medicines for Malaria Venture
